# Photothermal Performance of Metal–Phenolic Networks and Its pH-Dependent Coordination Regulation

**DOI:** 10.3390/molecules31101668

**Published:** 2026-05-15

**Authors:** Yuan Zou, Cheng Chang, Yuchen Xiu, Jingyan Liu, Fulin Yang, Can Liu, Yunwu Zheng, Xu Lin, Defa Hou

**Affiliations:** 1 National Joint Engineering Research Center for Highly-Efficient Utilization Technology of Forestry Resources, Southwest Forestry University, Kunming 650224, China; yzou24@swfu.edu.cn (Y.Z.); changc@swfu.edu.cn (C.C.); x3325655957yc@126.com (Y.X.); liujingyan_hit@126.com (J.L.); yangfulin0309@163.com (F.Y.); liucanswfu@163.com (C.L.); zyw85114@163.com (Y.Z.); linxunefu@126.com (X.L.); 2 State Key Laboratory of Advanced Polymer Materials, Sichuan University, Chengdu 610065, China

**Keywords:** metal–phenolic networks, Fe^3+^–gallic acid complex, photothermal conversion, pH-responsive coordination

## Abstract

Fe^3+^–polyphenol coordination complexes have attracted growing interest for photothermal applications due to their tunable chemistry and good biocompatibility. However, how pH and the metal-to-ligand ratio collectively affect their photothermal performance remains poorly understood. In this work, we synthesized Fe^3+^–gallic acid (GA) metal–phenolic networks (MPNs) under a wide range of pH conditions and different mixing ratios. The materials were then characterized through electron microscopy, infrared spectroscopy, UV-vis absorption, and photothermal testing. Our results show that a near-neutral pH (around 7) is critical for forming an effective ligand-to-metal charge transfer complex, which appears as a distinct absorption band near 560 nm. Acidic or strongly alkaline environments severely disrupt coordination and weaken light absorption. Among all formulations, the sample prepared at pH 7 with a suitable Fe^3+^/GA ratio gave the best photothermal conversion, reaching a temperature rise of 42.8 °C and a photothermal conversion efficiency of 32.67%. We also found that photothermal heating increases steadily with GA concentration and peaks sharply at neutral pH. These findings demonstrate that optimal photothermal efficiency requires both neutral pH and a well-balanced metal-to-ligand ratio. This work provides a simple and practical set of conditions for developing high-performance Fe^3+^-GA MPNs for applications such as local heating, antibacterial surfaces, and light-triggered drug release.

## 1. Introduction

Metal–phenolic networks (MPNs) have recently emerged as a versatile class of coordination materials, self-assembled from metal ions and natural polyphenols [1]. Thanks to the strong chelation between phenolic hydroxyl/carboxyl groups and transition metal ions (e.g., Fe^3+^, Cu^2+^), MPNs inherit excellent adhesion, biocompatibility, and pH-responsive coordination dynamics [2,3,4,5]. Among these systems, the Fe^3+^–gallic acid (GA) complex stands out. GA is a simple and abundant polyphenol. It forms stable complexes with Fe^3+^ under mild conditions, showing broad UV-vis-NIR absorption and good photothermal conversion capability [6]. These properties have enabled applications in surface functionalization, drug delivery, and photothermal therapy [7,8,9,10,11,12,13]. A key feature of Fe^3+^-GA MPNs is their pH-dependent coordination configuration, which directly tunes their electronic structure and optical absorption [14,15,16,17]. However, controlling the balance between coordination state and photothermal performance remains tricky. The network is highly sensitive to both the Fe^3+^/GA molar ratio and the solution pH. A systematic understanding of how these parameters affect light absorption and heat generation is still lacking.

Many efforts have tried to tailor the optical properties of Fe^3+^–polyphenol systems. Adjusting the pH during complexation shifts the deprotonation equilibrium of phenolic hydroxyls, thereby promoting or suppressing ligand-to-metal charge transfer [18,19,20,21,22]. Changing the metal-to-ligand ratio can also alter the dominant coordination mode and the absorption band [23]. For instance, it is known that under strongly acidic conditions protonation inhibits coordination, while under alkaline conditions excessive deprotonation or Fe(OH)_3_ precipitation weakens the charge transfer effect [24,25,26,27]. Despite these insights, most studies have looked at isolated parameter changes. A comprehensive evaluation of the combined effects of pH and molar ratio on photothermal conversion efficiency (η) is rare. Moreover, the influence of polyphenol concentration on the heat-generating capability of Fe^3+^-MPNs has not been quantitatively assessed. In our preliminary work, we compared Fe^3+^ complexes with two common polyphenols, tannic acid (TA) and GA, under identical conditions. The GA system consistently showed stronger light absorption and better photothermal heating. This led us to focus on GA for a detailed investigation.

In this work, we systematically synthesized Fe^3+^-GA MPNs under a range of controlled pH conditions and metal-to-ligand ratios, and we also varied the GA concentration. Our goal is to fill the gap between known coordination chemistry and photothermal performance by systematically mapping how pH, molar ratio, and concentration collectively determine the conversion efficiency. Using scanning electron microscopy, FT-IR, and UV-vis absorption spectroscopy, we found that the coordination interaction is highly pH-sensitive. Only near-neutral pH allows an effective charge transfer complex to form; acidic or strongly alkaline conditions severely suppress it. Notably, the sample prepared at neutral pH with a suitable Fe^3+^/GA ratio gave the highest photothermal performance, delivering a temperature rise of 42.8 °C and a conversion efficiency of 32.67%. Photothermal heating increased with GA concentration and peaked sharply at neutral pH. These results establish that optimal photothermal efficiency requires both neutral pH and a well-balanced metal-to-ligand ratio. Our work provides an optimal condition for photothermal conversion in the Fe^3+^-GA system, which may be useful for local heating, antibacterial surfaces, and light-controlled drug release.

## 2. Results

### 2.1. Structural Characterization of Fe^3+^-GA Complex

#### 2.1.1. Surface Morphology Characterization

**Figure 1:**
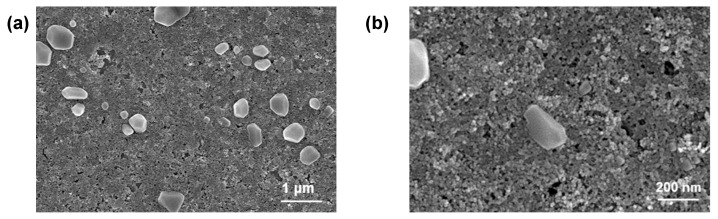
Structural characterization of Fe^3+^-GA complex. SEM of Fe^3+^-GA coating on PVDF: (**a**) 1 μm; (**b**) 200 nm.

In order to evaluate the surface morphology of the prepared Fe^3+^-GA coating, especially the morphological characteristics and size uniformity, the surface morphology of the material was characterized using a scanning electron microscope (SEM, ZEISS GeminiSEM 300, Oberkochen, Germany). As shown in Figure 1a,b, in the SEM image with a 1 μm scale, the Fe^3+^-GA composite coating loaded on PVDF film presents a nearly spherical or quasi-spherical particle morphology, and irregular crystal faces or pore characteristics can be observed at some particle edges. Furthermore, in the high-power SEM image at a scale of 200 nm, the particle surface is evenly distributed, with small size and good consistency, and the overall surface of the particle is relatively flat. The above results show that the prepared Fe^3+^-GA coating has good coatability and microstructure uniformity.

#### 2.1.2. Infrared Spectrum Characterization

**Figure 2:**
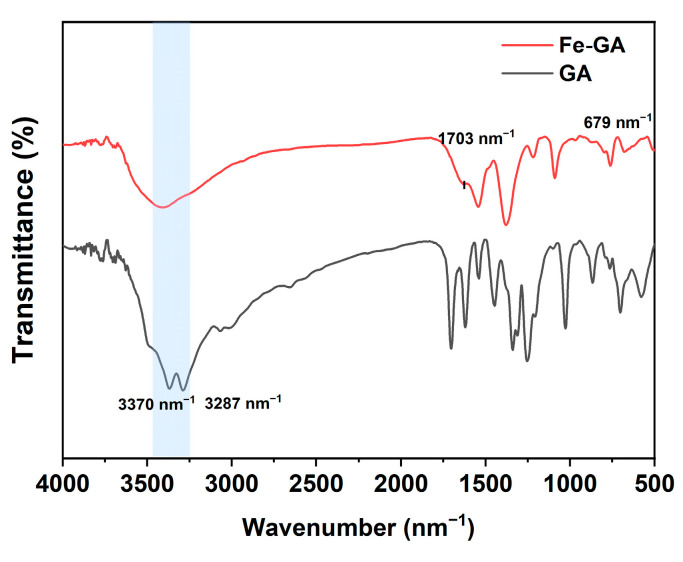
FTIR spectra of GA and Fe^3+^-GA.

The chemical composition of the prepared Fe^3+^-GA composite was analyzed using an infrared spectrometer (FT-IR, SHIMADZU IRXross, Kyoto, Japan) (Figure 2). Compared with GA alone, the Fe^3+^-GA composite showed some significant changes. At 3284 cm^−1^ and 3371 cm^−1^, the characteristic peaks of the stretching vibration and bending vibration of hydroxyl groups (-OH) on the benzene ring are obviously reduced and widened, which indicates that the number of phenolic hydroxyl groups (-OH) is reduced. The stretching vibration peak of C=O at 1703 cm^−1^ also weakened, which reflected the coordination reaction between GA and Fe^3+^. This may be because Fe^3+^ forms a coordination bond with a hydroxyl (-OH) or carboxyl (-COOH) group of GA, thus prolonging the conjugated network, which leads to a change in the vibration modes of these functional groups and the red shift in the peak.

#### 2.1.3. UV-Vis Spectrum Characterization

**Figure 3:**
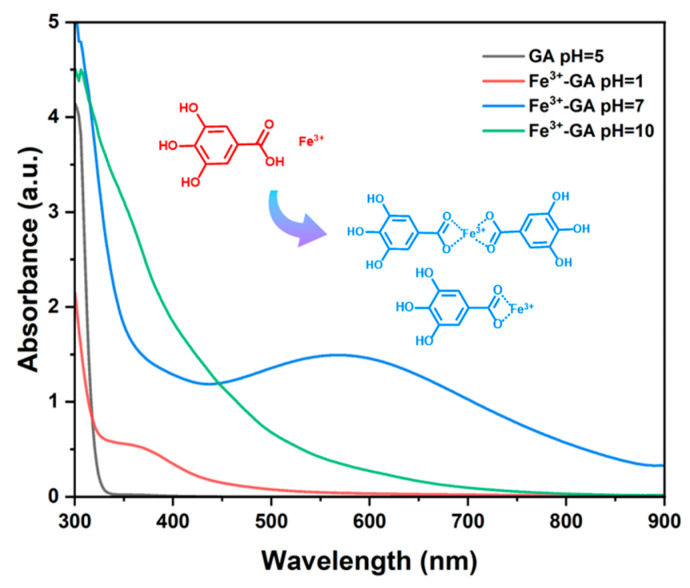
UV-vis absorbance spectra of GA (pH = 5) and Fe^3+^-GA (pH = 1, 7, 10).

The results of the ultraviolet–visible absorption spectrum analysis using a UV-vis spectrometer (UV-vis, SHIMADZU, Kyoto UV-2600, Japan) show that the Fe^3+^-GA complex formed under the condition of pH = 7 has a maximum absorption band at about 560 nm in the visible region (Figure 3). This band is consistent with a ligand-to-metal charge transfer (LMCT) transition, as previously reported for similar Fe^3+^–catecholate complexes. In contrast, the Fe^3+^-GA complex formed at pH = 1 has no similar absorption band. This observation is consistent with the expected protonation of phenolic hydroxyl and carboxyl groups under strongly acidic conditions, which prevents effective coordination. A likely explanation is that the phenolic hydroxyl and carboxyl groups on the GA molecule are highly protonated in a strong acidic environment; it is difficult to effectively coordinate with Fe^3+^, and the free Fe^3+^ and uncoordinated GA molecules mainly exist in the system. However, under the condition of pH = 10, the absorption band of the Fe^3+^-GA complex shifts blue. This shift may be due to the formation of Fe^3+^ (OH) precipitate induced by a high concentration of OH. At the same time, the strong alkaline environment promotes the oxidative degradation of GA, thus changing the coordination structure and weakening the LMCT. While direct spectroscopic evidence for these species was not obtained here, the pH-dependent trend is well explained by established coordination chemistry.

### 2.2. Evaluation of Light Absorption Performance

#### 2.2.1. Color Comparison and Analysis

**Figure 4:**
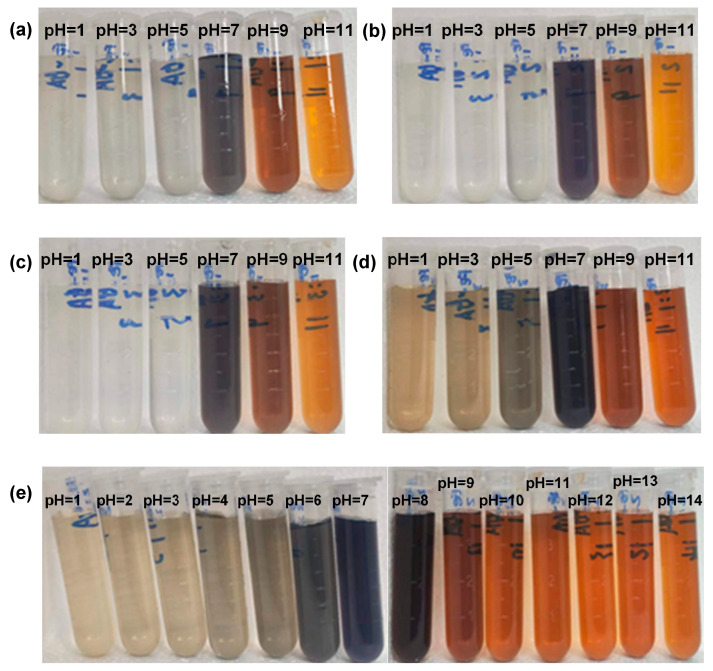
Optical picture of Fe^3+^-GA complex. (**a**–**e**) Comparison of the colors with different pH values at 1:1, 1:2, 1:3, 3:1 and 2:1 molar ratios, respectively.

In order to systematically study the response behavior of the coordination state in MPNs to external conditions, optical images of Fe^3+^-GA complexes under different preparation conditions were collected (Figure 4). The experimental conditions include the following: the molar ratio of Fe^3+^ to GA is increased from 1:3 to 3:1 in turn, and the pH of the reaction system is continuously adjusted from acidic to alkaline at each fixed molar ratio. As shown in Figure 3, a consistent trend was observed under all Fe^3+^/GA molar ratios: when pH < 7, the Fe^3+^-GA complex was colorless and transparent in water; when pH > 7, the color of the complex changes from black to purple with the increase in pH, and then it appears yellow. This remarkable color change shows that there is a coordination interaction between Fe^3+3+^ ions and GA molecules, and this interaction is highly sensitive to pH. With the increase in pH, the degree of deprotonation of phenolic hydroxyl groups is enhanced, which promotes the formation of different coordination configurations (such as single coordination, double coordination or triple coordination) between Fe^3+^ and GA, thus systematically regulating the electronic transition energy level of the complex and finally reflecting the obvious change in visible light absorption characteristics.

#### 2.2.2. Comparison and Analysis of UV-Vis Characterization

**Figure 5:**
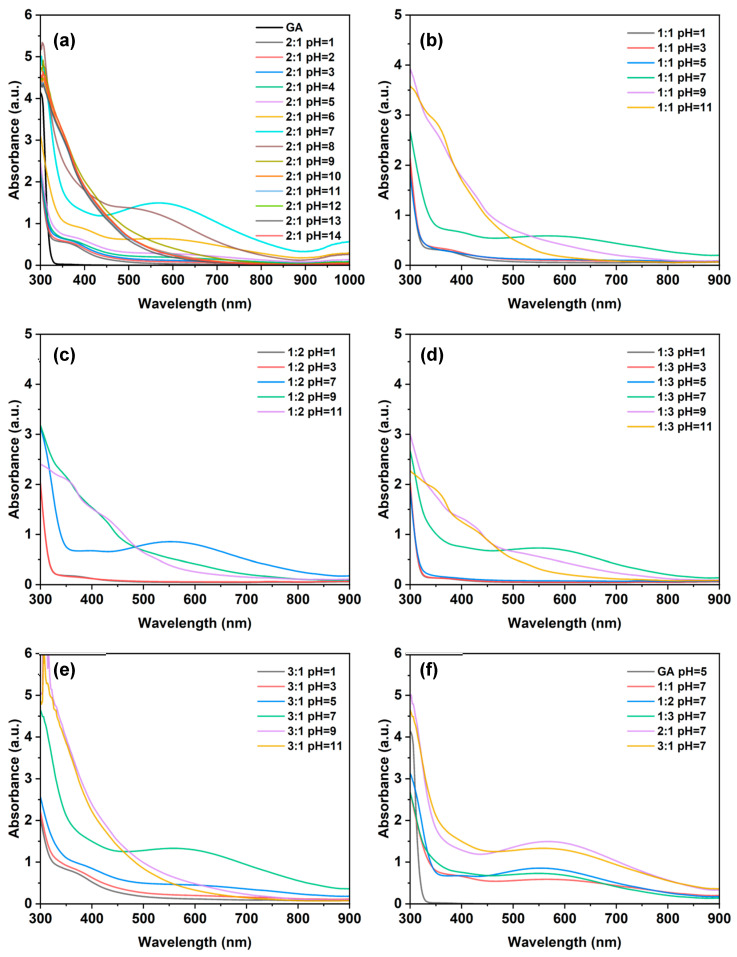
UV-vis absorbance spectra of Fe^3+^-GA complex. (**a**–**e**) Comparison of different pH absorption values at 1:1, 1:2, 1:3, 3:1 and 2:1 molar ratios, respectively. (**f**) Comparison of absorption values of different molar ratios at the same pH.

Further combined with ultraviolet–visible (UV-vis) absorption spectrum analysis (Figure 5a–e), it is found that, under the same Fe^3+^/GA molar ratio and different pH conditions, all samples with pH < 7 have almost zero absorption in the visible region (about 380–780 nm), and their spectral characteristics are similar to those of gallic acid (GA) alone. In contrast, when pH > 7, the absorption of each sample in the visible region is significantly enhanced. Specifically, when pH = 7, the absorption spectrum shows the widest absorption band and reaches the maximum absorption value at λ = 552 nm. However, when the pH further increases to the alkaline range (pH > 7), the absorption band gradually narrows and the peak absorption intensity decreases.

Generally speaking, the five Fe^3+^-GA MPN systems with different Fe^3+^/GA molar ratios all showed similar pH-dependent absorption behaviors: The overall absorbance of the system showed an upward trend under the condition of near neutral pH. Under acidic conditions (pH < 7), the absorbance increases with the increase in pH. However, in alkaline conditions (pH > 7), the absorbance gradually decreases with the increase in pH. In addition, by comparing five groups of samples with different molar ratios at the same pH (Figure 5f), it can be shown that the Fe^3+^-GA MPN with a molar ratio of Fe^3+^:GA of 2:1 shows high light absorption performance. Especially at pH = 7, the absorbance of the sample is significantly higher than that of other mixing groups in the whole visible light wavelength range from 380 nm to 780 nm, showing excellent overall absorption performance.

The above results show that the visible light absorption of Fe^3+^-GA MPN is enhanced under the conditions. A plausible interpretation is that this enhancement results from an extension of the conjugated system in the GA-Fe^3+^ complex, which, compared with single GA molecules, may capture more photons and thus improve the light absorption capacity. However, we cannot rule out that aggregation or concentration effects might also contribute to the observed spectral changes, as our current UV-vis data alone do not allow us to separate these factors. At low pH, most phenolic hydroxyl groups on GA molecules are protonated, which is expected to lead to competitive chelation between H^+^ and Fe^3+^ and likely inhibits the effective coordination between Fe^3+^ and GA. However, at high pH, the degree of deprotonation of GA molecules is too high, which probably affects its proper coordination and binding ability with Fe^3+^, leading to the decrease in the structural stability of the complex and the decrease in absorbance. We present these as hypotheses consistent with known coordination chemistry.

### 2.3. Photothermal Performance Evaluation

**Figure 6:**
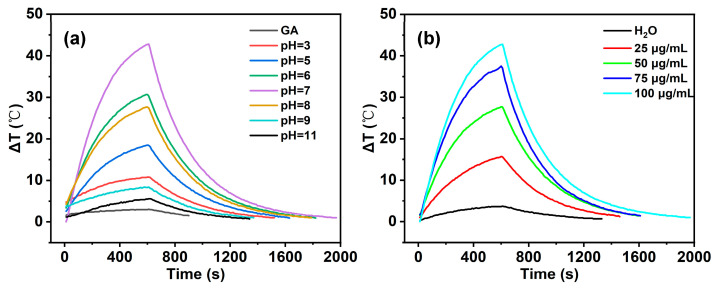
The temperature changes of Fe^3+^-GA MPN (100 μg/mL) irradiated by 808 nm laser at 2 W/cm^2^ for 10 min and then after the laser was shut off: (**a**) Fe^3+^-GA MPN with PH gradient change; (**b**) Fe^3+^-GA MPN with concentration gradient change.

To systematically evaluate the photothermal heating performance of Fe^3+^-MPN materials under different conditions, we performed the following experiments (Figure 6). Briefly, 1 mL of each sample and 1 mL of deionized water (as control) were injected into a cuvette. Each sample was then irradiated with an 808 nm near-infrared (NIR) laser for 10 min at a power density of 2.0 W/cm^2^. The laser spot was a circular area with a diameter of 1 cm. During irradiation, a thermocouple recorded the temperature change in real time at 10 s intervals. As a control, pure deionized water showed a negligible temperature rise of less than 3.5 °C under the same irradiation.

**Figure 7:**
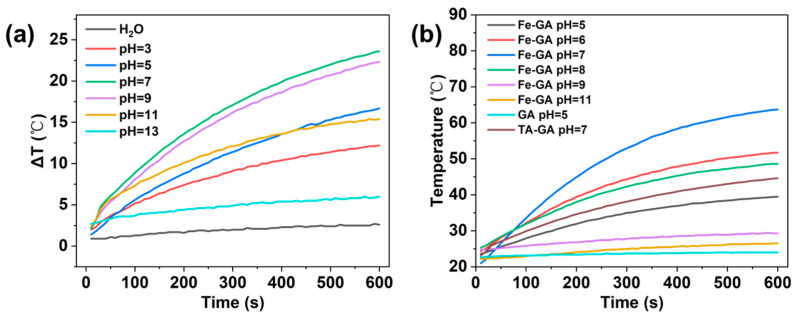
The temperature changes of Fe^3+^-TA MPN (100 μg/mL) irradiated by 808 nm laser at 2 W/cm^2^ for 10 min and then after the laser was shut off: (**a**) Fe^3+^-TA MPN with PH gradient change; (**b**) comparison of the Fe^3+^-GA and Fe^3+^-TA system (pH 7, 100 μg/mL, 2:1 molar ratio).

As shown in Figure 7a, the Fe^3+^-TA MPN system exhibited clear pH-dependent photothermal behavior, but the overall temperature rise was modest. We then compared Fe^3+^-GA and Fe^3+^-TA under identical conditions (pH 7, 100 μg/mL, 2:1 molar ratio) (Figure 7b). The GA system consistently gave a higher temperature increase and faster heating rate. This suggests that GA forms a more efficient photothermal complex with Fe^3+^ than TA does. Therefore, we chose the Fe^3+^-GA system for all subsequent detailed investigations.

#### 2.3.1. Effect of pH on Photothermal Properties

According to the analysis in Figure 6b, with the change in pH value, the photothermal temperature rise range (ΔT) of the system also changes regularly. When pH = 7, ΔT is the largest; when the pH value deviates from neutrality (whether in the direction of acidity or alkalinity), ΔT gradually decreases. This result shows that the macroscopic photothermal effect of the Fe^3+^-GA network is significantly regulated by the pH value. Under neutral conditions (pH = 7), the coordination structure is likely in its most stable state, as inferred from the UV-vis absorption maximum at 560 nm and the highest photothermal efficiency, which is conducive to the formation of efficient non-radiation relaxation channels, thus showing the maximum photothermal conversion efficiency and temperature rise range.

To exclude the influence of ionic strength, we performed a control experiment by adding NaCl to the Fe^3+^-GA sample at pH 7 to match the ionic strength of the pH 13 sample. The photothermal heating curves showed no significant difference compared to the unmodified pH 7 sample (Figure S2), confirming that the observed pH dependence is primarily due to changes in the protonation state of GA rather than ionic strength.

#### 2.3.2. Effect of Concentration on Photothermal Performance

In this study, the effect of complex concentration on the photothermal properties of Fe^3+^-GA MPN was further investigated. The molar ratio of Fe^3+^/GA in Fe^3+^-GA-7 system was fixed at 2:1, and pH was 7. Samples with GA concentrations of 25 μg/mL, 50 μg/mL, 75 μg/mL and 100 μg/mL were prepared, respectively, and the samples were irradiated continuously for 10 min under an 808 nm laser (2.0 W/cm^2^). As shown in Figure 7, the photothermal performance of Fe^3+^-GA MPN is positively correlated with its concentration: with the gradual increase in the concentration of GA in the solution, the temperature rise range (ΔT) of the system gradually increases, showing obvious concentration dependence. The results show that the concentration of GA has a significant regulatory effect on the photothermal properties of the Fe^3+^-GA MPN. In a certain concentration range, the higher the concentration of GA, the faster the heating rate of the solution, the higher the final temperature and temperature difference that can be achieved, and the corresponding photothermal conversion ability will be enhanced. This phenomenon can be attributed to the fact that the increase in GA concentration promotes the formation density of the Fe^3+^-GA coordination structure and increases the number of light absorption groups in the system, thus improving the efficiency of light energy capture and the thermal effect caused by non-radiation relaxation.

#### 2.3.3. Calculation of Photothermal Conversion Efficiency

The photothermal conversion efficiency is calculated based on the time-dependent temperature variation. The photothermal conversion efficiency (*η*) of Fe^3+^-GA MPN is calculated as follows:

$$\eta =\frac{hs\left(T_{Max}-T_{surr}\right)-Q_{dis}}{I\left(1-10^{-A}\right)}$$

where *h* is the heat transfer coefficient, *s* is the irradiation area, and *T_Max_* represents the highest temperature of the solution in a 1.5 mL cuvette after near-infrared irradiation for 10 min. *T_surr_* is the ambient temperature, *Q_dis_* is the heat loss, *I* is the laser power (2.0 W/cm^2^), and *A* is the absorbance of the Fe^3+^-GA MPN at 808 nm. The value of *h_s_* is calculated using:

$$hs=\frac{m_{d}C_{d}}{\tau_{s}}$$

*m_d_* is the mass of the sample (1 g), *C_d_* represents the heat capacity of the water solvent (4.2 J^−1^g^−1^K^−1^), and τ*_s_* is the time constant.

$$t=-\tau_{s}\ln\theta$$

**Figure 8:**
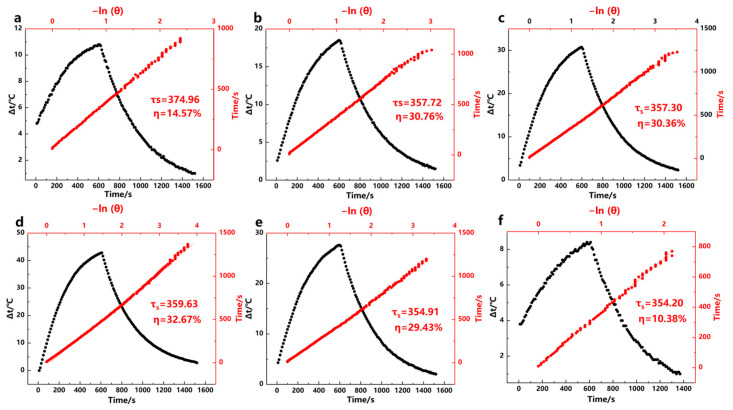
Temperature rise and fall curves of Fe^3+^-GA complex (black dots) and the ratio of −lnθ to linear time (red dots). (**a**) pH = 3, (**b**) pH = 5, (**c**) pH = 6, (**d**) pH = 7, (**e**) pH = 8, and (**f**) pH = 10.

**Figure 9:**
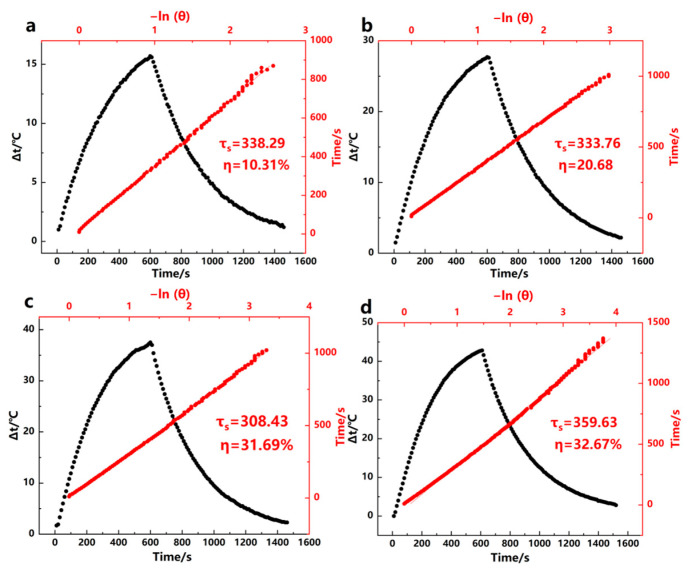
Temperature rise and fall curves of Fe^3+^-GA complex (black dots) and the ratio of −lnθ to linear time (red dots). (**a**) 25 μg/mL, (**b**) pH = 50 μg/mL, (**c**) pH = 75 μg/mL, and (**d**) pH = 100 μg/mL.

Based on the above evaluation results of photothermal performance, in this study, Fe^3+^-GA samples (Fe^3+^-GA MPN-3, Fe^3+^-GA MPN-5, Fe^3+^-GA MPN-6, Fe^3+^-GA MPN-7, Fe^3+^-GA MPN-8, Fe^3+^-GA MPN-10,) and four groups of different GA concentrations (Fe^3+^-GA MPN25, Fe^3+^-GA MPN50, Fe^3+^-GA MPN75, Fe^3+^-GA MPN100) were further studied. Based on the natural cooling curves of each sample under 808 nm laser irradiation, the photothermal properties of Fe^3+^-GA MPN were quantitatively verified, as shown in Figure 8 and Figure 9. Relevant calculation results are summarized in Table 1. The relative errors for ΔT and η are typically within 5% of the mean values in triplicate experiments, indicating good reproducibility and measurement precision. The photostability of the optimal Fe^3+^-GA sample was examined through three consecutive on/off cycles of 808 nm laser irradiation. As shown in Figure S1, the temperature rise remained nearly unchanged over the three cycles, indicating good photostability under the tested conditions.

## 3. Discussion

Our results clearly show that the photothermal performance of Fe^3+^-GA MPNs is delicately controlled by pH and the metal-to-ligand ratio. The UV-vis data reveal that a strong absorption band appears only near neutral pH, characteristic of ligand-to-metal charge transfer (LMCT) [7,28,29]. At a low pH, the protonation of phenolic groups prevents chelation, leaving the solution nearly transparent. At a high pH, excessive deprotonation or Fe(OH)_3_ precipitation disrupts coordination, consistent with earlier reports on Fe^3+^–polyphenol systems. So why does a neutral pH work best? Just above pH 7, GA gradually deprotonates, forming bis- or tris- complexes with Fe^3+^. These higher-order coordination modes extend the conjugated network and enhance non-radiative decay, which is exactly what we see in the photothermal measurements.

Our preliminary comparison between GA and tannic acid (TA) is also worth noting. GA is much smaller and has fewer adjacent hydroxyl groups per molecule. Yet, in our hands, Fe^3+^-GA consistently outperformed Fe^3+^-TA under identical conditions. One key factor is the large difference in molecular weight: TA is about 1700 g/mol, while GA is only about 170 g/mol. At the same mass concentration, the molar concentration of GA is roughly ten times higher than that of TA. Thus, even though each TA molecule carries more phenolic groups, the total number of GA molecules per volume is much greater, leading to a higher density of effective coordination sites for Fe^3+^. This may be because GA’s compact structure allows a higher density of coordination sites, leading to stronger LMCT and better heat generation. In addition, TA’s bulky and highly branched structure tends to cause steric hindrance and aggregation, which further reduces the accessibility of its phenolic groups for Fe^3+^ binding. Similar trends have been observed when comparing simple and polymeric polyphenols [30,31]. We also found that photothermal heating increases with GA concentration, which makes sense: more GA molecules mean more absorbing chromophores per unit volume. The optimal sample (2:1 Fe^3+^/GA at pH 7) gave an efficiency of 32.67%, suggesting a stable bis- complex that balances binding strength and electron delocalization. Taken together, our findings show that, for the Fe^3+^-GA system, neutral pH and a moderate excess of GA relative to Fe^3+^ yield the best photothermal response. This insight should be useful for developing polyphenol-based photothermal materials for biomedical and coating applications.

## 4. Materials and Methods

### 4.1. Reagents

Hydrochloric acid (HCl) solution (37%), sodium hydroxide (NaOH) (96%), ferric chloride hexahydrate (FeCl_3_·6H_2_O) (99%), tannic acid (TA) (99%), and gallic acid (GA) (99%) were all purchased from Shanghai Adamas Reagent Co., Ltd. (Shanghai, China).

### 4.2. Synthesis of Fe^3+^-GA MPN

A 10 mM gallic acid solution and 30 mM ferric chloride (FeCl_3_·6H_2_O) solution were prepared. After mixing the two solutions, the solution immediately turned black. The complex network structure can be changed by adjusting the pH of the solution with 1 M HCl and 1 M NaOH. The prepared samples are summarized in Table 2.

### 4.3. Preparation FG-MPN Coating

A 10 mM GA solution and 30 mM ferric chloride (FeCl_3_) solution are respectively prepared. The prepared PVDF was immersed in GA aqueous solution (10 mL) for 1 h. Then, the FeCl_3_ solution was injected into the above solution to rapidly generate deep purple; the pH was adjusted to 7 with 1 M NaOH solution so that the reaction solution was changed from suspension to solution, and the coordination reaction was continued for 8 h to obtain FG-MPN coating. After washing with excess deionized water, FG-MPN coating was freeze-dried and stored in a drying oven for further use.

## 5. Conclusions

In summary, this work demonstrates that the photothermal performance of Fe^3+^-GA metal–phenolic networks is governed by pH and the metal-to-ligand ratio in a predictable manner. We acknowledge that the underlying coordination chemistry (pH-dependent LMCT) is not new. What is new is the systematic, quantitative mapping of how three parameters, including pH, the metal/ligand ratio, and GA concentration, jointly influence the photothermal conversion efficiency. Our initial comparison between tannic acid and gallic acid revealed that GA consistently forms more efficient photothermal complexes with Fe^3+^. The systematic characterization of Fe^3+^-GA MPNs then showed that near-neutral pH is essential for establishing an effective ligand-to-metal charge transfer complex. Acidic or strongly alkaline conditions severely disrupt coordination and weaken light absorption. Under optimal conditions (neutral pH and a suitable Fe^3+^/GA ratio), the material achieved a temperature rise of 42.8 °C and a photothermal conversion efficiency of 32.67%. We also found that photothermal heating increases with GA concentration and peaks sharply at neutral pH. Therefore, rather than overstating novelty, we propose this work as a useful empirical optimization for Fe^3+^-GA MPNs. These findings establish a clear structure–property relationship for Fe^3+^-GA MPNs. More broadly, our work offers a simple pH-based optimization for the Fe^3+^-GA system. Whether similar conditions apply to other polyphenol–metal systems remains to be investigated, with potential applications in local heating, antibacterial surfaces, and light-controlled systems.

## Figures and Tables

**Table 1:** Summary of maximum temperature, temperature increase, and photothermal conversion efficiency for various samples.

Sample	A (808)	T_Max_ (°C)	ΔT_Max_ (°C)	*η*
Fe^3+^-GA MPN-3	0.11	25.0 ± 0.5	6.0 ± 0.5	14.6 ± 1.1
Fe^3+^-GA MPN-5	0.14	35.0 ± 0.9	16.0 ± 0.9	30.8 ± 1.2
Fe^3+^-GA MPN-6	0.28	46.3 ± 1.1	27.3 ± 1.1	30.4 ± 1.0
Fe^3+^-GA MPN-7	0.50	61.8 ± 1.4	42.8 ± 1.4	32.7 ± 1.3
Fe^3+^-GA MPN-8	0.23	42.3 ± 1.0	23.3 ± 1.0	29.4 ± 1.1
Fe^3+^-GA MPN-10	0.13	24.0 ± 0.4	5.0 ± 0.4	10.4 ± 0.7
Fe^3+^-GA MPN25	0.59	33.7 ± 0.7	14.7 ± 0.7	10.3 ± 0.6
Fe^3+^-GA MPN50	0.47	45.2 ± 1.1	26.2 ± 1.1	20.7 ± 0.9
Fe^3+^-GA MPN75	0.39	54.8 ± 1.3	35.8 ± 1.3	31.7 ± 1.2
Fe^3+^-GA MPN100	0.5	61.8 ± 1.3	42.8 ± 1.3	32.7 ± 1.1

**Table 2:** Summary of sample codes, initial Fe^3+^/GA molar ratios, GA concentrations, and pH values used for preparing Fe^3+^-GA metal–phenolic networks.

Sample (*i* = pH)	Initial Fe^3+^/GA Molar Ratio	GA Concentration	pH
Fe^3+^-GA MPN(A)-*i*	1:3	100 μg/mL	1, 3, 5, 7, 9, 11, 13
Fe^3+^-GA MPN(B)-*i*	1:2		
Fe^3+^-GA MPN(C)-*i*	1:1		
Fe^3+^-GA MPN-*i*	2:1		
Fe^3+^-GA MPN(E)-*i*	3:1		
Fe^3+^-GA MPN25	2:1	25 μg/mL	7
Fe^3+^-GA MPN50		50 μg/mL	
Fe^3+^-GA MPN75		75 μg/mL	

## Data Availability

The original contributions presented in this study are included in the article. Further inquiries can be directed to the corresponding authors.

## Supplementary Materials

The following supporting information can be downloaded at: https://www.mdpi.com/article/10.3390/molecules31101668/s1, Figure S1: Temperature curves of Fe^3+^-GA MPN-7 under three on/off cycles and under 808 nm laser irradiation; Figure S2: Photothermal heating curves of Fe^3+^-GA MPN at pH 7 without (control) and with additional NaCl (ionic strength matched to pH 13 sample).
