# Supplementary material for: Photothermal Performance of Metal–Phenolic Networks and Its pH-Dependent Coordination Regulation
Source: Molecules. 2026 May 15;31(10):1668. doi: 10.3390/molecules31101668 (PMC13209588; doi:10.3390/molecules31101668)
Supplement: Supplementary file 1 [file molecules-31-01668-s001.zip › molecules-4293962-supplementary.pdf]

Support Information

Photothermal Performance of Metal–Phenolic Networks and Its pH-Dependent Coordination Regulation

Yuan Zou [1,2], Cheng Chang [1], Yuchen Xiu [1], Jingyan Liu [1], Fulin Yang [1], Can Liu [1], Yunwu Zheng [1], Xu Lin [1] and Defa Hou [1,*]

[1] National Joint Engineering Research Center for Highly-Efficient Utilization Technology of Forestry Resources, Southwest Forestry University, Kunming 650224, China
[2] State Key Laboratory of Advanced Polymer Materials, Sichuan University, Chengdu 610065, China
* Correspondence: houdefa001@163.com

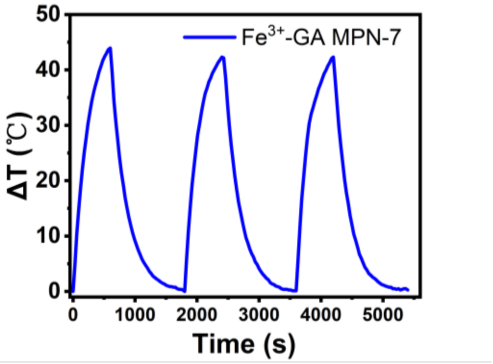

Figure S1. Temperature curves of Fe^{3+}-GA MPN-7 under three on/off cycles and under 808 nm laser irradiation.

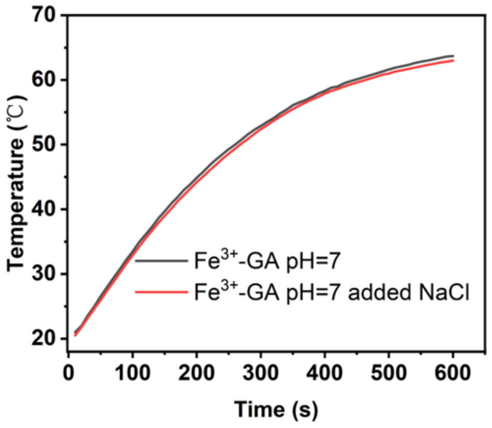

Figure S2. Photothermal heating curves of Fe^{3+}-GA MPN at pH 7 without (control) and with additional NaCl (ionic strength matched to pH 13 sample).
